# Lifetime cost-effectiveness and equity impacts of the Healthy Primary School of the Future initiative

**DOI:** 10.1186/s12889-020-09744-9

**Published:** 2020-12-09

**Authors:** Marije Oosterhoff, Eelco A. B. Over, Anoukh van Giessen, Rudolf T. Hoogenveen, Hans Bosma, Onno C. P. van Schayck, Manuela A. Joore

**Affiliations:** 1 Department of Clinical Epidemiology and Medical Technology Assessment (KEMTA), Maastricht University Medical Center MUMC+/ Care and Public Health Research Institute (CAPHRI), Maastricht University, P.O. Box 5800, 6202 AZ Maastricht, The Netherlands; 2 Centre for Nutrition, Prevention and Health Services, National Institute of Public Health and the Environment, Bilthoven, the Netherlands; 3 Expertise Center for Methodology and Information Services, National Institute for Public Health and the Environment, Bilthoven, the Netherlands; 4 Department of Social Medicine, Care and Public Health Research Institute (CAPHRI), Faculty of Health, Medicine and Life Sciences, Maastricht University, Maastricht, The Netherlands; 5 Department of Family Medicine, Care and Public Health Research Institute (CAPHRI), Faculty of Health, Medicine and Life Sciences, Maastricht University, Maastricht, The Netherlands

**Keywords:** Cost-effectiveness, Health impact modelling, Lifestyle prevention, Childhood obesity

## Abstract

**Background:**

This study estimated the lifetime cost-effectiveness and equity impacts associated with two lifestyle interventions in the Dutch primary school setting (targeting 4–12 year olds).

**Methods:**

The Healthy Primary School of the Future (HPSF; a healthy school lunch and structured physical activity) and the Physical Activity School (PAS; structured physical activity) were compared to the regular Dutch curriculum (*N* = 1676). An adolescence model, calculating weight development, and the RIVM Chronic Disease Model, calculating overweight-related chronic diseases, were linked to estimate the lifetime impact on chronic diseases, quality adjusted life years (QALYs), healthcare, and productivity costs. Cost-effectiveness was expressed as the additional costs/QALY gained and we used €20,000 as threshold. Scenario analyses accounted for alternative effect maintenance scenarios and equity analyses examined cost-effectiveness in different socioeconomic status (SES) groups.

**Results:**

HPSF resulted in a lifetime costs of €773 (societal perspective) and a lifetime QALY gain of 0.039 per child versus control schools. HPSF led to lower costs and more QALYs as compared to PAS. From a societal perspective, HPSF had a cost/QALY gained of €19,734 versus control schools, 50% probability of being cost-effective, and beneficial equity impact (0.02 QALYs gained/child for low versus high SES). The cost-effectiveness threshold was surpassed when intervention effects decayed over time.

**Conclusions:**

HPSF may be a cost-effective and equitable strategy for combatting the lifetime burden of unhealthy lifestyles. The win-win situation will, however, only be realised if the intervention effect is sustained into adulthood for all SES groups.

**Trial registration:**

Clinicaltrials.gov (NCT02800616). Registered 15 June 2016 – Retrospectively registered.

**Supplementary Information:**

**Supplementary information** accompanies this paper at 10.1186/s12889-020-09744-9.

## Background

Physical inactivity, unhealthy diets, overweight, and obesity are among the leading risk factors for the burden of disease in many countries worldwide [1, 2]. Before 1990, less than 35% of Dutch adults were overweight or obese, which has increased to more than 50% in 2018 [3]. This trend is burdensome, because excess weight is a risk factor for (early) development of chronic diseases and concomitant quality of life losses, premature death, and costs [4–7]. School-based lifestyle interventions are viewed as a promising strategy to reduce the overweight-related burden as attitudes and behaviours can be changed more easily as compared to later stages in life [8–10].

Insight on the short, medium, and long-term impacts on health, healthcare costs and costs in other sectors is essential to inform implementation and investment decisions on school-based lifestyle interventions. In addition to an overall cost-effectiveness outcome, decision-makers should be informed on how this estimate changes under different circumstances and choices. Firstly, there is uncertainty on the persistence of the intervention effect from childhood into early adulthood [11, 12]. Secondly, school-based lifestyle interventions may not only impact on healthcare cost but may also affect productivity in later life. Productivity impacts are, however, not always included in cost-effectiveness studies on these interventions. Thirdly, there is a high need for interventions that reduce (or at least do not widen) the disparities in health outcomes between people with a high and low socioeconomic position. So far, it seems that lifestyle interventions can either increase or decrease health inequities between different socioeconomic groups [13, 14]. As a consequence, trade-offs between cost-effectiveness and equity can occur (e.g. an intervention is cost-effective but high SES group benefits more as compared to low SES groups). Cost-effectiveness estimates for different socioeconomic status (SES) groups can be used to inform these potential trade-offs [15].

In the Southern region of the Netherlands, two new healthy school environments have been introduced which aimed at enhancing health promotion in the Dutch primary school setting (corresponding to 4–12 year olds) [16]. The Healthy Primary School of the Future (HPSF) included a daily healthy school lunch and mid-morning snack and a daily structured physical activity program, while the Physical Activity School (PAS) focused on the structured physical activity program only. The implementation process and the 2-year (cost-) effectiveness have been previously examined [16–19]. In the current study, the lifetime cost-effectiveness and equity impacts of the interventions were compared to the regular school curriculum. Cost-effectiveness was assessed from a healthcare and societal perspective. Scenario analyses were undertaken to assess the influence of alternative effect maintenance scenarios and equity analyses to examine cost-effectiveness in different socioeconomic groups.

## Methods

The methods and results of this economic evaluation were reported according to the Consolidated Health Economic Evaluation Reporting Standards (CHEERS) Statement (see checklist in Additional File 1) [20].

### Strategies

The ‘Healthy Primary School of the Future’ initiative aimed to integrate health promotion in the Dutch primary school setting, which starts at 4 years of age until 12 years of age [16]. Three strategies were compared:

1. **Healthy Primary School of the Future (HPSF).** A healthy morning snack and daily healthy lunches were provided in combination with structured sessions including sports, play, and creative activities. To facilitate the implementation of activities the school day was extended with about 30 min [16].
2. **Physical Activity School (PAS).** This school environment targeted physical activity only by offering the structured sessions including sports, play, and creative activities during the lunch break.
3. **Regular school curriculum in Dutch primary schools.** Control schools maintained the normal school curriculum (no interventions).

In a quasi-experimental study, HPSF (2 schools) and PAS (2 schools) were followed from 2015 to 2019, and compared to 4 control schools from the same region (Parkstad region, Dutch Province of Limburg) [16]. Bartelink et al. (2019) analysed the 2-year effects of HPSF and PAS versus control schools on dietary and physical activity behaviour, and body mass index (BMI) z-score [17, 19]. These effects are based on all participants who were enrolled at schools from baseline onwards (and had a two-year intervention exposure) and participated in at least one of the measurement waves (*N* = 1676, control: *N* = 661, HPSF: *N* = 537, PAS = 478). See Additional File 2 for the baseline characteristics of the study sample.

### Cost-effectiveness modelling

A lifetime horizon was adopted, starting at age 4. Two periods were distinguished: 1) childhood and adolescence and 2) adulthood. The lifetime health and cost impacts of HPSF and PAS were modelled through the changes in BMI development. For children receiving the regular school curriculum, it was assumed that BMI and weight status did only change depending on age. Outcomes were expressed in quality-adjusted life years (QALYs), which combines the impact on health-related quality of life (HRQOL) and length of life. Cost-effectiveness was assessed from a healthcare perspective and societal perspective (including productivity costs).

#### Childhood and adolescence

The childhood and adolescence model covered the period from 4 up to 20 years of age. The health state transition model consisted of three weight categories: normal weight, overweight, and obesity. The model was implemented in Microsoft Excel 2010 with a cycle length of one year.

Information on children’s BMI development was obtained from a previous study (Oosterhoff et al: BMI trajectories after primary school-based lifestyle intervention: unravelling an uncertain future. A mixed methods study: submitted). In this study, BMI values were extrapolated until 20 years of age. Based on the 2-year effect on BMI z-score, it was calculated that the effects of HPSF and PAS on BMI were − 0.21 kg/m^2^ [95% CI: − 0.38; − 0.05] (HPSF) and − 0.17 kg/m^2^ [95% CI: − 0.33; 0.00] (PAS) as compared to control schools [17]. The entire trajectory was lowered, assuming that the observed relative intervention effects were fully maintained until 20 years of age (reference scenario: constant intervention effects). BMI values were converted into weight categories by using the Dutch reference values for skewness and variation and the age- and sex-specific international cut-off points for childhood overweight and obesity [21, 22].

The costs associated with delivery of HPSF and PAS were previously estimated [23]. The intervention costs were the sum of material costs and time investments. The costs reflected a ‘steady state’, representing the costs for routine implementation in daily practice [24]. Costs were updated to 2018 prices using consumer price indexes [25]. Given a healthcare perspective, the intervention costs amounted to €4.47 [HPSF] and €2.16 [PAS] per child per day. At HPSF, the school day was extended with 30 min per day (4 days a week) to offer extra time for the lunch and the PA program. Parents/caregivers could spent this time on (un) paid work. Considering a societal perspective, the value of the extended school day at HPSF lowers the net intervention costs of HPSF (net societal opportunity costs) (€0.96 [HPSF] per child per day) (see Additional File 3). School absenteeism days were selected as indicator of productivity in childhood. The excess missed school days associated with overweight and obesity were obtained from the literature and were combined with the number of missed schooldays for children with a healthy weight and the Dutch shadow price for school absenteeism [16, 26, 27]. Information on HRQOL weights and healthcare costs (general practitioner and specialist visits) days for normal weight, overweight, and obese children and adolescents were obtained from the literature (see Additional File 3). Inputs collected with the quasi-experimental study were used in sensitivity analyses as they were derived from a relatively small number of children (4%, 44 children were obese at baseline). Costs and QALYs were calculated for each age-cohort between 4 and 12 years of age, and were aggregated based on the number of Dutch 4–12 year-olds (2019) to calculate the results for a school cohort. Detailed information on model inputs and assumptions can be found in Table 1 and Additional File 3.

**Table 1:** Key model input parameters

Input parameters	Mean value	95% CI	Distribution (se)	Data source and assumptions ^e^
Population estimates	Number of boys and girls aged 4 until 12 years of age			CBS Statline.
***Intervention effect estimate***				Bartelink et al. (2019)
Relative effect after 2 years of intervention in children aged 4–12 years	**BMI z-score**			Assumption: full effect maintenance over lifetime ^a^
	HPSF: −0.083	[−0.15;-0.02]	Gamma (0.08)	
	PAS: −0.066	[−0,13;-0.00]	Gamma (0.09)	
	**BMI (standard deviation 2.55 kg/m**^**2**^**)**			
	HPSF: −0.21	[− 0.38;-0.05]	Gamma (0.08)	
	PAS: −0.17	[−0.33;-0.00]	Gamma (0.09)	
SES-specific 2-year relative effects	**BMI z-score**			Bartelink et al. (2019). Converted to BMI effects with standard deviation of 2.55 (based on the study sample at baseline).
	HPSF vs control			
	low SES: −0.103	[−0.22;-0.02]	Gamma (0.16)	
	middle SES: −0.049	[−0.16;-0.06]	Gamma (0.14)	
	high SES: −0.063	[−0.18;-0.05]	Gamma(0.15)	
	PAS vs control			
	low SES: −0.067	[− 0.18;-0.05]	Gamma (0.15)	
	middle SES: −0.056	[−0.18;-0.06]	Gamma (0.16)	
	high SES: −0.051	[−0.16;-0.06]	Gamma (0.14)	
***Effect maintenance scenarios***				Oosterhoff et al. (2020)
1.Constant- and decreasing effects after primary school Maintenance factor uncontrolled environment	HPSF: 0.22	[0.04;0.39]	Lognormal (0.09)	
	PAS: 0.22	[0.06;0.37]	Lognormal (0.08)	
2. Increasing- and decreasing effects after primary school Relative BMI effect with household multiplier				
	HPSF: −0.30	[−0.42;-0.18]	Gamma(0.06)	
	PAS: −0.19	[−0.27;-0.12]	Gamma (0.04)	
3. Increasing effects Maintenance factor household maintainer				
	HPSF: 1.67	[1.48;1.85]	Lognormal (0.09)	
	PAS: 1.10	[1.01;1.19]	Lognormal (0.05)	
***Intervention cost estimate***				Oosterhoff et al. (2019)
Net intervention costs, societal perspective^b^	HPSF: €153 per year (€0.96 per day) (2016)		Fixed	
	PAS: €346 per year (€2.16 per day) (2016)		Fixed	
Net intervention costs, healthcare perspective ^b^	HPSF: €715 per year (€4.47 per day) (2016)		Fixed	
**Childhood and adolescence**				
***Weight status***				
Cut-off values of overweight and obesity (kg/m^2^)			Fixed	Cole et al. (2000)
BMI distribution Dutch children	Age and sex-specific values for skewness and variation			Schönbeck et al. (2011)
***Health-related quality of life***				
Utility weights	Normal weight: 0.85	[0.84;0.87]	Beta (0.01)	Brown et al. (2018)
	Overweight: 0.83	[0.81;0.85]	Beta (0.01)	
	Obesity: 0.82	[0.79;0.84]	Beta (0.01)	
***Health resource use***				
Average number of GP visits / year	59.6% children visiting GP * 6.7 visits / year		Fixed	Statline (n.d.)
Average number of specialist visits / year	27.0% children visiting GP * 9.7 visits / year		Fixed	Statline (n.d.)
Ratio of HC costs for overweight vs. normal weight	1		Fixed	Gortmaker et al. [based on Table A.3.2]
Ratio of HC costs for obesity vs. normal weight	1.22	[1.21;1.22]	Lognormal (0.00)	
Cost price per GP visit ^b^	€34		Fixed	Zorginstituut Nederland (2015)
Cost price per specialist visit ^b^	€94		Fixed	Zorginstituut Nederland (2015)
***School absenteeism***				
Median number of school absenteeism days / year ^c^	5.0		Gamma (3.26)	Additional analysis based on data collection as described by Willeboordse et al. (2016)
Ratio of absenteeism for overweight vs. normal weight	1.27	[1.03;1.56]	Lognormal (0.14)	An et al. (2017)
Ratio of absenteeism for obesity vs. normal weight	1.54	[1.33;1.78]	Lognormal (0.11)	
Cost price per school absenteeism day ^b^	€27		Fixed	Drost et al. (2014)
**Adulthood**				
Weight status ^a^	Normal weight, overweight, obesity		Log-odds	Fifth Dutch Growth Study. Schönbeck et al. (2009)
Chronic diseases ^d^	**Obesity related diseases**: acute myocardial infarction, coronary heart disease, stroke, renal, colorectal, breast, prostate, and endometrium cancer, diabetes mellitus, hip, knee arthritis, and low back pain. **Indirect-related diseases:** Chronic obstructive pulmonary disease, lung, stomach, esophagus, larynx, bladder, pancreas, and oral cavity cancer		Prevalence: log-oddsIncidence: lognormal	RIVM Chronic Disease Model. Hoogenveen et al. (2010), van Baal et al. (2006)
**Adulthood**				
***Health-related quality of life***				
Utility weights (for chronic disease)			Fixed	Dutch Burden of Disease Study. Melse et al. (2000)
***Health resource use & unit costs***				
Disease healthcare costs			Fixed	Dutch Cost of Illness Study. Slobbe et al. (2006)
***Productivity costs***				
Sick leave days	Overweight women: 3.64		Fixed	Lehnert et al. (2014)
	Overweight men: 0			
	Obese women: 5.19			
	Obese men: 3.48			
Net labour participation	72.2%		Fixed	CBS Statline (2017)
Working hours per week	31.4 (6.28 per day / 5 days a week)		Fixed	CBS Statline (2017)
Productivity costs / hour ^b^	€36		Fixed	Zorginstituut Nederland (2015)

**Notes:** *BMI z-score* Body mass index standardized score, *CI* Confidence interval, *GP* General practitioner, *HC* Healthcare, *HPSF* The Healthy Primary School of the Future, *HRQOL* Health-related quality of life, *PAS* The Physical Activity School, *QALY* Quality-adjusted life year

^a^ In the adulthood model, the uncertainty of the intervention effect was incorporated by including the overweight and obesity prevalence rates at young adulthood as probabilistic parameters. This uncertainty parameter reflected the boundaries of the 95% confidence interval of the intervention effect on BMI. The overweight and obesity prevalence rates at 20 years of age were included as multivariate normal distributions with a perfect correlation

^b^ Updated to 2018 prices

^c^ The analysis was based on crossectional data (baseline year). Regression analysis with a Poisson distribution was used to reflect the count data. The effect of weight status (normal weight [reference level], overweight and obesity) on school absenteeism days was analysed. Analysis were additionally adjusted for sex, grade, school type, socioeconomic status and ethnicity

^d^ We used coupled sets of random draws for the prevalence, incidence and mortality for the chronic diseases in the probabilistic sensitivity analysis

^e^ References can be found in Additional File 3

#### Adulthood

The RIVM Chronic Disease Model (CDM) was used for projecting effects from 20 years of age until the cohort reached the age of 100 years [28]. This probabilistic health economic model with the Markov property estimates the prevalence, incidence, and mortality of chronic diseases based on changes in risk factors. The model was built in R version 3.5.1. In the current study, the proportions of weight categories (normal weight, overweight, and obesity) were used to estimate the lifetime incidence of obesity-related chronic diseases and other diseases during the life years gained. Utilities, healthcare costs, and mortality were dependent on the prevalence of chronic diseases. Information on the RIVM CDM and the key inputs can be found in Additional File 4 and Table 1. Cost data were indexed to the Dutch 2018 price level using consumer price indexes. Productivity losses in adulthood were incorporated through the relation between weight category up to 67 years of age (the current age of retirement in the Netherlands) and the number of annual sick leave days from work as reported by Lehnert et al. (2014) [29].

### Analyses

The per child health effects (QALYs) and costs for the childhood and adolescence period and the adulthood period were summed up. Costs and effects were adjusted for the differences in time at which they occur with a discount rate of 4 and 1.5% per year, respectively, according to the Dutch guidelines for costing in economic evaluations [30]. Incremental cost-effectiveness ratios (ICERs) were calculated by dividing the change in costs between the alternatives by the change in QALYs. Under a healthcare perspective, the change in costs between the strategies was determined by the intervention costs and the differences in healthcare costs. For the societal perspective, the differences in school absenteeism costs and productivity costs in adulthood were also included. Interventions were considered to be cost-effective if the ICERs did not exceed the willingness to pay thresholds of €20.000 per QALY gained [31].

### Sensitivity and effect maintenance scenario analyses

Inputs were varied in univariate deterministic sensitivity analyses to explore the impact of input parameters on the results (Additional File 5). Probabilistic sensitivity analyses calculated the probability of cost-effectiveness in relation to the uncertainty in the input parameters (Additional File 5). For the childhood model, results were based on 500 draws per age cohort, whereas 100 random draws were used in the adulthood model. We performed scenario analyses for effect maintenance. In a previous study, we elicited expert opinions on the maintenance of intervention effects after the observed 2 year-period into young adulthood (20 years of age) (Oosterhoff et al: BMI trajectories after primary school-based lifestyle intervention: unravelling an uncertain future. A mixed methods study: submitted). This resulted in three potential pathways that were used in scenario analyses.

#### Scenario 1: constant- and decreasing effects after primary school

It was assumed that effects would remain constant with continued exposure during the primary school period. Effects would decay after primary school when the intervention exposure ends. This scenario corresponded to a relative BMI effect of − 0.04 kg/m^2^ (HPSF and PAS) at 20 years of age.

#### Scenario 2: increasing- and decreasing effects after primary school

It was assumed that parental involvement would lead to the uptake of behaviour changes by the household over time, leading to more favourable effects during the primary school period with continued exposure. Effects would decay after primary school when the intervention exposure ends. This scenario corresponded to a relative BMI effect of − 0.06 kg/m^2^ (HPSF) and − 0.04 kg/m^2^ (PAS) at 20 years of age.

#### Scenario 3. Increasing effects

It was assumed that intensive parental involvement would lead to the uptake of behaviour changes by the household. This would lead to sustained behavioural changes during and after the primary school period. This scenario corresponded to a relative BMI effect of − 0.50 kg/m^2^ (HPSF) and − 0.21 kg/m^2^ (PAS) at 20 years of age.

### Equity analyses

Children’s socioeconomic background was measured as the combination of maternal and paternal education level, and household income adjusted for household size, and was categorized in three groups (low, middle and high SES) [17]. Bartelink et al. (2019) reported that the effects of HPSF and PAS on BMI were slightly higher for children with a low SES as compared to children with a high SES (not statistically significant different) (Table 1) [17]. These SES-specific intervention effects on BMI were included in the childhood model. Equity impacts were represented by the difference in health outcomes between the low and high SES group. The relationship between equity impacts and cost-effectiveness was presented in an equity-efficiency impact plane, displaying the (potential) trade-off between cost-effectiveness and health equity [15]. To specify this trade-off we converted cost-effectiveness and health equity impacts to a health metric. The net health equity impact was expressed as the absolute difference in QALY gains between the high and low SES group. The net cost-effectiveness or net total health impact was calculated as follows: overall QALY gains – (difference costs* willingness to pay threshold). After two years of intervention, no statistical significant differences were found for the BMI-effects between SES groups [17]. It is, however, not clear if effects can be equally maintained in all SES groups. Experts indicated that effect maintenance throughout adolescence may be higher for children with a high SES background as compared to children with a low SES (Oosterhoff et al: BMI trajectories after primary school-based lifestyle intervention: unravelling an uncertain future. A mixed methods study: submitted). We therefore also compared the increasing effects scenario (until 20 years of age) for the high SES group to the constant and decreasing effects scenario for the low SES group.

## Results

**Fig. 1:**
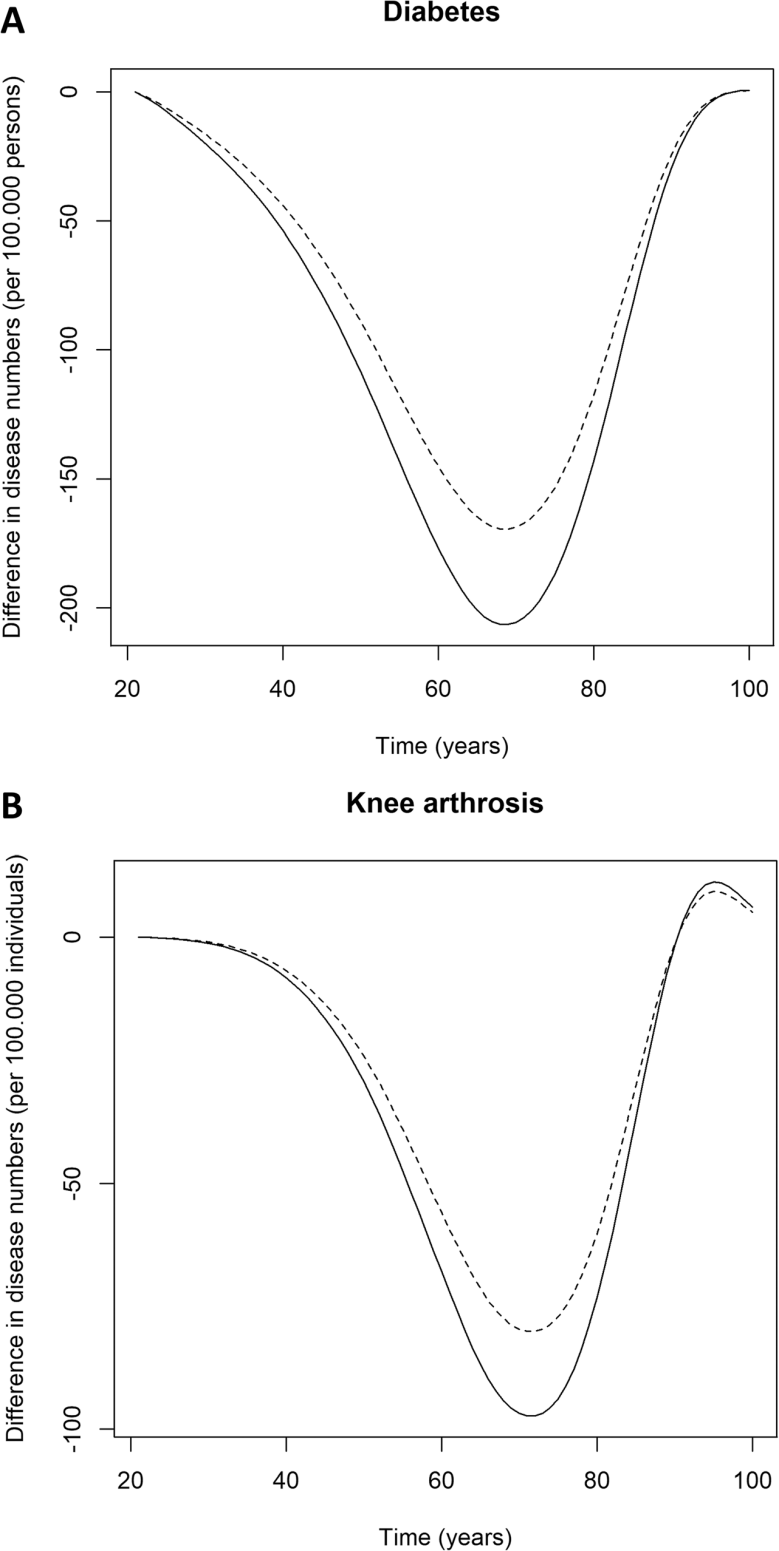
Differences in diabetes and knee arthritis prevalence numbers. **Notes:** Solid line: HPSF versus control schools. Dashed line: PAS versus control schools

Table 2 shows the health outcomes and costs. For illustrative purposes, Fig. 1 shows that from young adulthood onwards, HPSF and PAS resulted in a reduction of chronic diseases (e.g. diabetes and knee/hip arthrosis). The avoided cases of chronic diseases reached their maximum around 70 years of age. Subsequently, the differences in disease numbers declined because individuals that were exposed to HPSF and PAS lived longer and experienced chronic disease during these life years gained. The lifetime QALYs per child amounted to 52.164 (control schools), 52.196 (PAS) and 52.203 (HPSF). Under a healthcare perspective, the lifetime cost were €249,535 (control schools), €251,419 (PAS) and €253,175 (HPSF) per child. The lifetime cost per QALY gained amounted to €58,698 for PAS vs. the regular school curriculum. The lifetime cost per QALY gained was €248,206 for HPSF vs. the next best alternative (PAS). When adopting a societal perspective, the total cost were €259,380 (control schools), €260,152 (HPSF) and €261,025 (PAS). The lifetime cost per QALY gained was €19,734 for HPSF versus the regular school curriculum. HPSF was dominant over PAS, as PAS had a higher cost and a lower QALY gain as compared to HPSF (Table 3).

**Table 2:** Health effects and cost impacts of HPSF and PAS for the childhood and adulthood life span (per child)

Perspective	Deterministic results (discounted)	Control schools	PAS	HPSF
**Healthcare**	**Intervention costs** ^**a**^	–	€1587	€3279
	**Childhood and adolescence (4–20 years of age)**			
	Healthcare costs	€4855	€4854	€4854
	Years with overweight	2.718	2.569	2.532
	Years with obesity	0.396	0.371	0.365
	QALYs	12.803	12.806	12.807
	**Adulthood (20 years of age – death)**			
	Healthcare costs of obesity-related and indirect diseases	€244,680	€244,978	€245,043
	LYs	52.131	52.157	52.163
	QALYs	39.362	39.390	39.397
	**Lifetime**			
	Healthcare costs	€249,535	€249,832	€249,896
	QALYS	52.164	52.196	52.203
**Societal**	**Intervention costs (societal opportunity costs)** ^**a**^	–	€1587	€702
	**Childhood and adolescence (4–20 years of age)**			
	Productivity costs (school absenteeism)	€1813	€1808	€1807
	**Adulthood (20 years of age – death)**			
	Productivity costs (sick leave days)	€8031	€7798	€7748
	**Lifetime**			
	Productivity costs	€9844	€9606	€9554

**Notes:** *BMI* Body mass index, *HC* Healthcare, *HPSF* The Healthy Primary School of the Future, *LYs* Life years, *PAS* The Physical Activity School, *QALYs* Quality-adjusted life years. Costs discounted at 4% and effects discounted at 1.5% per year

^a^ The average intervention cost for a school cohort (targeting children from all primary school grades). For the intervention costs under the healthcare perspective, the productivity-related offsets due to the extended school day at HPSF were excluded (see main text)

**Table 3:** Expected lifetime cost-effectiveness results of HPSF and PAS

Strategies	Costs		Difference in costs		QALYs		Difference in QALYs		Difference in costs / Difference in QALYs
***Healthcare perspective***^a^									
Regular school curriculum	€249,535	[€225,176; €234,083]	–		52.164	[51.795; 52.667]	–		–
PAS	€251,419	[€226,968; €236,002]	€1883	[€1638; €2032]	52.196	[51.817; 52.725]	0.032	[0.008; 0.050]	€58,698
HPSF	€253,175	[€228,700; €237,822]	€1756	[€1491; €2109]	52.203	[51.820; 52.686]	0.007	[−0.021; 0.042]	€248,206
***Societal perspective***^b^									
Regular school curriculum	€259,380	[€233,506; €246,790]	–		52.164	[51.795; 52.667]	–		–
HPSF	€260,152	[€234,169; €247,664]	€773	[€525; €941]	52.203	[51.817; 52.725]	0.039	[0.017; 0.060]	€19,734
PAS	€261,025	[€235,065; €248,459]	€872	[€522; €1139]	52.196	[51.820; 52.686]	−0.007	[−0.042; 0.021]	Dominated

**Notes:** *HPSF* the Healthy Primary School of the Future, *LYs* Life years, *PAS* The Physical Activity School, *QALYs* Quality-adjusted life years. Between brackets: uncertainty interval based on the probabilistic results

^a^ Costs: intervention costs + healthcare costs

^b^ Costs: intervention costs + healthcare costs + school absenteeism costs

### Sensitivity and effect maintenance scenario analyses

The parameters in the childhood and adolescence model had little influence on the lifetime cost-effectiveness results (Table 4). When HRQOL in the adulthood model was determined by weight category instead of by chronic disease, the cost per QALY gained decreased. In contrast, a shorter time horizon, equal discounting for costs and effects (3.0%) as is common in many other countries instead of differential discounting (costs: 4.0%, effects: 1.5%), and short-term intervention cost instead of steady state cost drove up the cost-effectiveness results.

**Table 4:** Results of one-way deterministic sensitivity analyses on the lifetime cost per QALY gained

Time period	Parameters	Healthcare perspective		Societal perspective	
		PAS vs control schools		HPSF vs control schools	
	***Reference scenario*** (deterministic results: Total net cost / Total QALYs gained)	€58,698		€19,734	
		*lower*	*upper*	*lower*	*upper*
Childhood & adolescence	Two-year intervention effects (+/− 20%)	€57,581	€59,619	€19,319	€20,061
	Effect maintenance factors (+/− 20%)	€51,934	€62,020	€17,782	€20,977
	Intervention costs (+/− 20%)	€48,807	€68,588	€16,150	€23,317
	HRQOL weights (+/− 20%)	€57,546	€59,897	€19,338	€20,146
	Ratio of HC costs for overweight and obesity vs. normal weight (+/− 20%)	€58,649	€58,747	€19,683	€19,784
	Ratio of school absenteeism days for overweight and obesity vs. normal weight (+/− 20%)	–		€19,599	€19,868
	HRQOL based on the quasi-experimental study ^a^		€60,612		€20,390
	Ratio of HC costs for overweight and obesity vs. normal weight based on the quasi-experimental study	€58,603		€19,637	
	Ratio of school absenteeism days for overweight and obesity vs. normal weight based on the quasi-experimental study	–			€19,756
Adulthood	HRQOL determined by weight category instead of by chronic disease in adulthood	€36,397		€12,273	
	Sick leave days for overweight and obesity vs. normal weight (+/− 20%)	–		€18,285	€25,779
	Time horizon until age 70 years		€156,646		€79,523
	Inclusion on short-term intervention costs (year 1 and 2) instead of estimated long-run costs only		€67,423		€32,649
	Discount rates of 3% for both costs and effects		€183,687		€31,230

**Notes:** *HPSF* the Healthy Primary School of the Future, *PAS* the Physical Activity School

Costs discounted at 4% and effects discounted at 1.5% per year

^a^ We found no overall trend in utility decrements for overweight and obesity in the quasi-experimental study. Brown et al. (2018) also report that evidence on utility decrements in young children is inconclusive. No utility decrements were applied during the primary school period

**Fig. 2:**
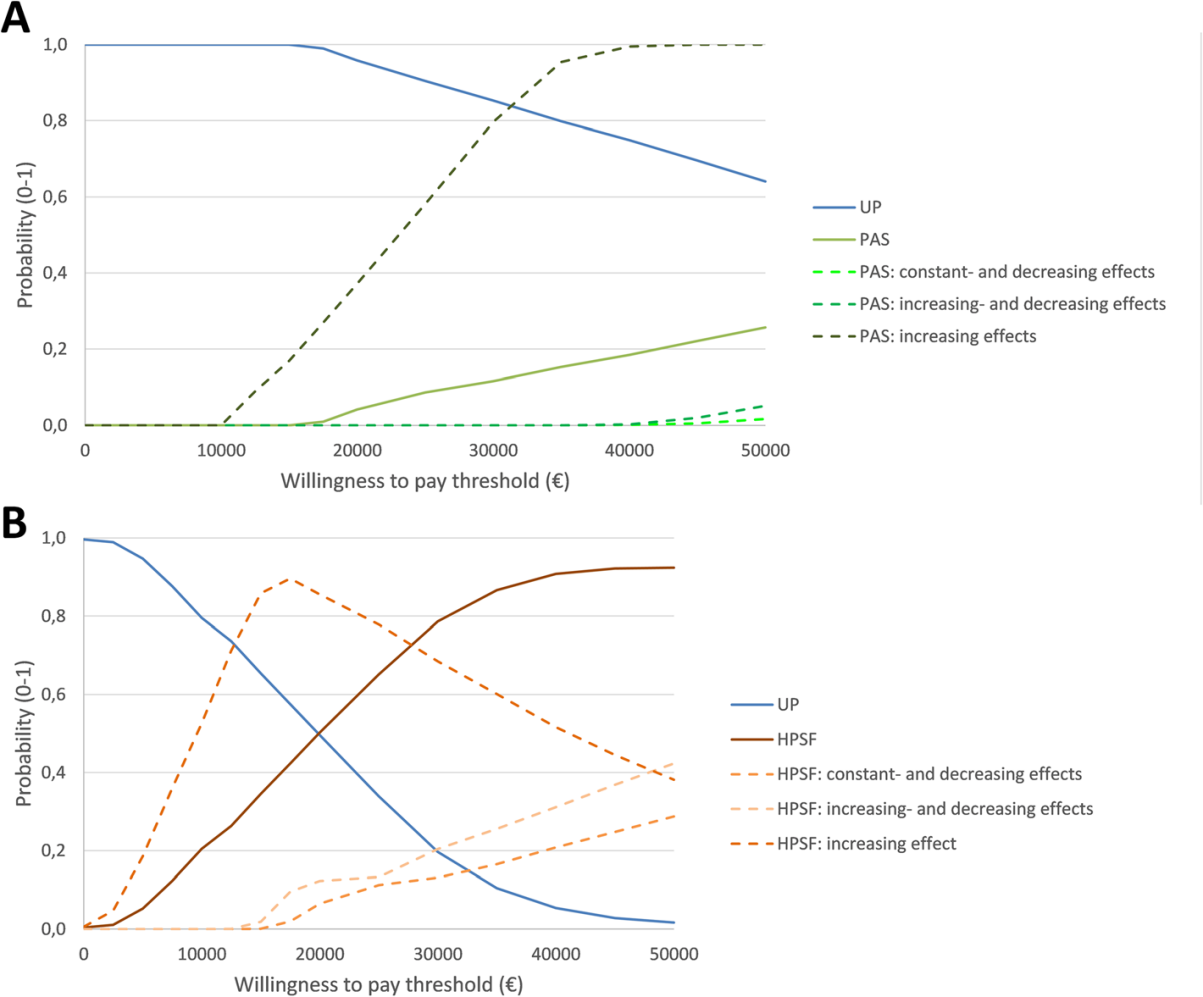
Probability of cost-effectiveness (cost-effectiveness acceptability curve). Panel A) Healthcare perspective. Panel B) Societal perspective. **Notes:** *HPSF * the Healthy Primary School of the Future, *PAS* the Physical Activity School, *UP* usual practice, regular school curriculum. Dashed lines represent the probabilitiy of cost-effectiveness for HPSF and PAS under the alternative scenarios. The probability of cost-effectiveness for UP in these scenarios is not presented

Under a healthcare perspective, the regular school curriculum had the highest probability of being cost-effective (Table 3, Fig. 2). For the societal perspective, HPSF had a 50% probability of being cost-effective at the €20,000 threshold (66% at the €25,000 threshold) (Fig. 2).

Cost-effectiveness was also assessed for alternative effect maintenance scenarios. The increasing effects scenario (assuming uptake of behaviour changes by the household) resulted in the lowest cost per QALY gained (Table 5 and Fig. 2). The cost per QALY gained under the healthcare perspective declined to €51,934 for PAS vs. control (reference: €58,698), but the regular school curriculum had the highest probability of being cost-effective. For a societal perspective, HPSF became cost-saving in comparison to the regular school curriculum with the increasing effects scenario (reference: €19,734 (Table 5) and HPSF had a 85% probability of being cost-effective.

**Table 5:** Results of subgroup and scenario analyses [deterministic results]

SES group	*All*	*Low*	*Middle*	*High*	*All*	*Low*	*Middle*	*High*
	PAS versus control schools				HPSF versus PAS			
**Healthcare perspective**	**€58,698**	€58,611	€59,583	€60,039	**€248,206**	€221,006	€295,330	€256,314
*Effect maintenance scenarios*								
Constant- and decreasing effects after primary school	€195,323	€194,768			€264,098			
Increasing- and decreasing effects after primary school	€167,761				€246,622			
Increasing effects	€51,934			€51,934	€257,803			
	HPSF versus control schools				HPSF versus PAS ^a^			
**Societal perspective**	**€19,734**	€19,237	€20,659	€20,265				
*Effect maintenance scenarios*								
Constant- and decreasing effects after primary school	€57,415	€54,808						
Increasing- and decreasing effects after primary school	€36,344							
Increasing effects	Dominant^a^			Dominant^a^				

**Notes:** *SES* Socioeconomic status, *QALYs* Quality-adjusted life years

^a^ HPSF led to cost-savings in comparison to the regular school curriculum

### Equity analyses

**Fig. 3:**
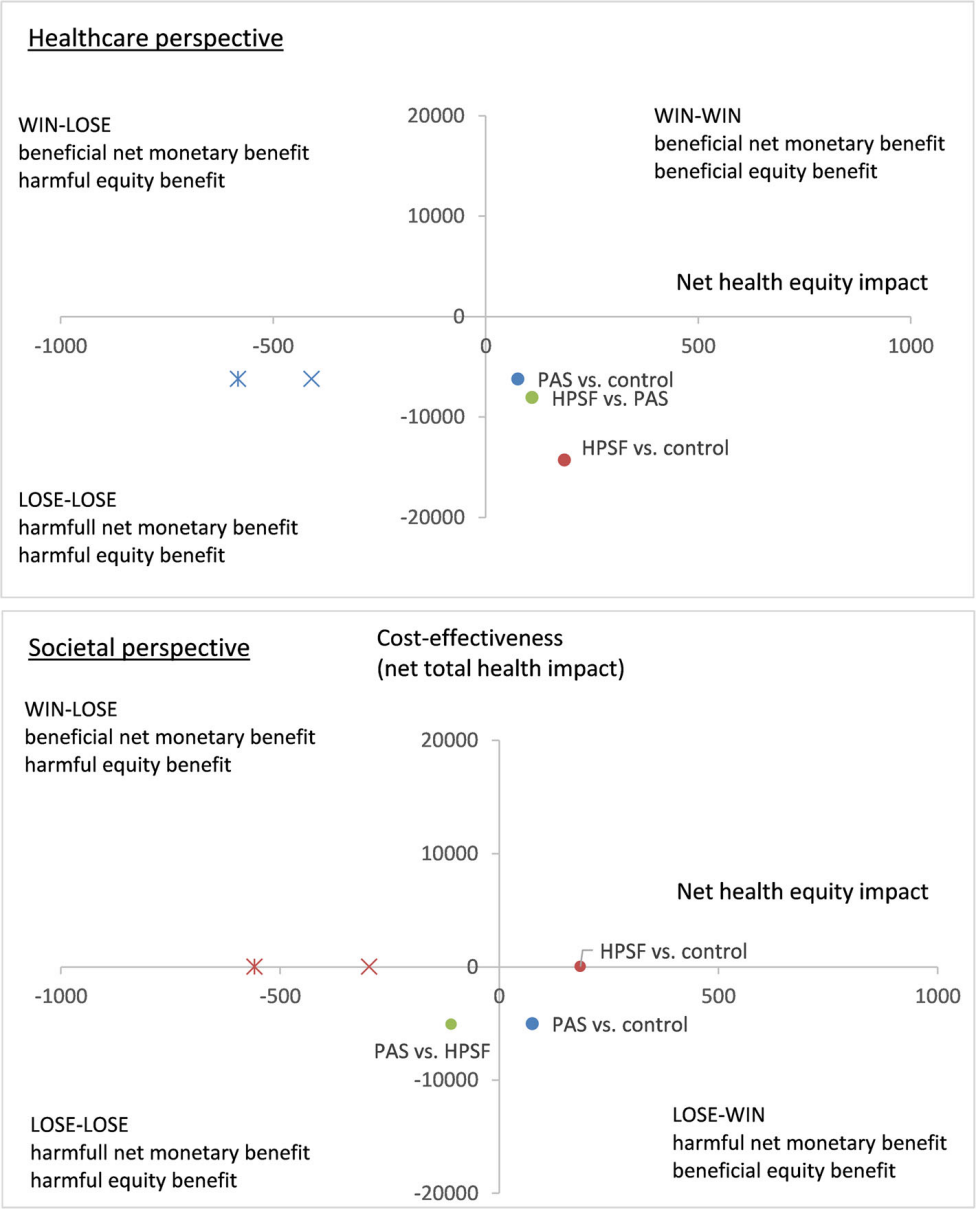
Equity-efficiency impact plane. **Notes**: *HPSF* the Healthy Primary School of the Future, *PAS* the Physical Activity School. Health impact expressed in QALYs per 100,000 persons. × = increasing intervention effects for the high socioeconomic group and constant intervention effects for the low socioeconomic group. * = increasing intervention effects for the high socioeconomic group and decreasing intervention effects for the low socioeconomic group

HPSF and PAS led to more health benefits and a lower cost per QALY gained in the low SES group as compared to the high SES group. Under a healthcare perspective, PAS fell in the lose-win quadrant of the equity-efficiency impact plane: the cost-effectiveness of PAS vs. the regular curriculum exceeded the willingness to pay threshold and had a harmful cost-effectiveness impact (− 6207 QALYs per 100,000 persons). On the other hand, PAS resulted in a beneficial equity impact (76 QALYs gained per 100,000 persons for the low versus high SES group). Under the societal perspective, HPSF fell in the win-win quadrant of the equity-efficiency impact plane: the cost-effectiveness of HPSF vs. the regular curriculum fell below the willingness to pay threshold and had a positive cost-effectiveness impact (52 QALYs gained per 100,000 persons). At the same time, HPSF also led to a positive equity impact (185 QALYs gained per 100,000 persons). Under the hypothetical situation that effects would only be maintained throughout adolescence in the high SES group (decreasing intervention effect scenario in low SES groups), HPSF vs. control schools would lead to a win-lose situation: a beneficial cost-effectiveness impact but a harmful health equity impact (297 and − 558 QALYs gained per 100,000 persons (Fig. 3).

## Discussion

Delivering HPSF and PAS to a cohort of primary-school based children (age 4–12 years) resulted in a lifetime QALY gain of 0.039 (HPSF vs. control schools) and 0.032 (PAS vs. control schools) per child. Under a healthcare perspective, the costs of HPSF and PAS per QALY gained exceeded the Dutch threshold value for prevention [31]. When additionally including the impacts on productivity (societal perspective), HPSF was marginally cost-effective in comparison with PAS and the regular curriculum (HPSF vs. regular curriculum: €19,734 per QALY gained; had a 50% probability of being cost-effective; HPSF was dominant compared to PAS). In addition, HPSF had a favourable health equity impact in comparison to the regular school curriculum (more QALY gains for the low versus the high SES group). This win-win situation did, however, not apply if effects decayed after the primary school period or if effects were only maintained in high SES groups.

The findings show that the future health and cost impacts of HPSF and PAS are greatly influenced by the in/exclusion of productivity costs (1), and the assumptions pertaining to the maintenance of the intervention effect (2). The additional costs of HPSF (HPSF vs control schools) were lower under the societal perspective than under the healthcare perspective. This was due to the impact of the extended school day on productivity of parents, of excess weight on school absenteeism, and of morbidity on productivity in later life. Other studies also showed that cost-effectiveness outcomes for school health promotion are substantially lower under a societal perspective, and some interventions even become cost-saving [32, 33]. Including productivity impacts may be relevant in order to gain a full insight into the societal benefits of school health promotion, but it remains unclear whether these impacts are considered important for decision-making on these interventions.

Two assumptions were made regarding the maintenance of the intervention effect: 1) effect maintenance during childhood until young adulthood (20 years), and 2) effect maintenance during adulthood. In the main analysis it was assumed that the observed effects were fully maintained into young adulthood (constant relative effects). The scenario analyses showed that the cost-effectiveness results were sensitive to the assumptions on the effect maintenance until adulthood. In a previous study, expert elicitation revealed that intervention effects could only be maintained into adulthood if the changes in healthy eating and physical activity behaviours are be adopted by the household (Oosterhoff et al: BMI trajectories after primary school-based lifestyle intervention: unravelling an uncertain future. A mixed methods study: submitted). Intensive parental involvement is likely required for this so-called effect transfer. Some effect decay, may be therefore likely. Follow-up measurements (without additional interventions) in secondary school could be undertaken to examine the effect maintenance and reduce the uncertainty around future benefits. The feasibility, costs, and effects of additional actions such as intensive parental involvement (particularly for low SES families) and intervention continuation in secondary schools could be examined to foster future impacts. The effects of the primary-school based lifestyle interventions in adulthood were modelled by adjusting the proportion of normal weight, overweight, and obesity at 20 years of age (see adulthood model). The probabilities of moving from one weight category to another were not adjusted, which implied that the relative intervention effect fades out over the lifetime (e.g. a person eventually moves to the same weight category as in the usual practice situation).

We included the SES-specific BMI-effects of HPSF and PAS and calculated the corresponding lifetime impacts. The main findings showed that the intervention effects and cost-effectiveness outcomes were in favour of the low SES group (reference scenario). It is not known whether the (unobserved) effect maintenance will differ between SES groups. Considering expert beliefs that effects may be only maintained in high SES groups, a trade-off between cost-effectiveness and health equity will occur. The calculations were based on the presumption that SES does not vary over time, because we did not have detailed information on the tracking of children’s socioeconomic position into young adulthood. In reality, children may obtain a different SES position as compared to their parents, induced by the current educational and labour opportunities. We expect that this applies to children from both low and high SES groups (with equal relative differences), as the relative differences in primary school advices in the Netherlands have been stabilized [34]. We, therefore, think that the dynamic character of children’s SES may not greatly affect the estimated lifetime equity impact. Monitoring of the dynamic character of SES is, however, advisable to test this assumption, and to contribute to the inclusion of SES in population health models.

We assessed the cost-effectiveness of HPSF and PAS for a school cohort of 4–12 year old children. Alternatively, an age-cohort such as all 8-year old children (average age) school could have been selected which led to a lower cost per QALY gained. We felt that modelling the impact for a school cohort was most in line with the quasi-experimental study, which focused on assessing effectiveness instead of efficacy. Furthermore, we included HRQOL weights related to chronic diseases and excess weight. Further studies on the HRQOL effects of excess weight in different subgroups (e.g. age and sex) could contribute to a more precise estimation of the health impacts of obesity prevention programs. Last, it should be noted that the intervention effects of HPSF and PAS were based on the effects after two years of implementation. We are currently examining the effects and cost-effectiveness after the four-year intervention period.

## Conclusions

Given the societal benefits and the Dutch threshold for prevention, HPSF is a marginally cost-effective strategy for combatting the lifetime burden associated with unhealthy lifestyles when assuming constant relative effects. In addition, HPSF has the potential to reduce health inequalities over the lifespan. Implementation is, however, associated with uncertainty: HPSF will not result in a win-win situation if effects fade out during adolescence and/or effects are only maintained among children with a high socioeconomic background. It is therefore paramount to enact upon the uncertain effect maintenance by means of follow-up measurements and by exploring the value of additional interventions.

## Supplementary Information

**Additional file 1.**

**Additional file 2.**

**Additional file 3.**

**Additional file 4.**

**Additional file 5.**

## Abbreviations

BMI: Body mass index
CDM: Chronic Disease Model
CI: Confidence interval
HPSF: Healthy Primary School of the Future
HRQOL: Health-related quality of life
ICER: Incremental cost-effectiveness ratio
PAS: the Physical Activity School
QALY: Quality-adjusted life year
SES: Socioeconomic status
