# Supplementary material for: Lifetime cost-effectiveness and equity impacts of the Healthy Primary School of the Future initiative
Source: BMC Public Health. 2020 Dec 9;20:1887. doi: 10.1186/s12889-020-09744-9 (PMC7724829; doi:10.1186/s12889-020-09744-9)
Supplement: Supplementary file 2 — Additional file 2. [file 12889_2020_9744_MOESM2_ESM.docx]

Additional File 2. Characteristics of the study sample at baseline (T0) [17].

|  |  | **Total (N=1676)** | |  | **HPSF (N=537)** | **PAS (N=478)** | **Control (N=661)** |
| --- | --- | --- | --- | --- | --- | --- | --- |
|  |  | ***N*** | ***Missing (%)*** | ***% / Mean (±SD)*** | ***% / Mean (±SD)*** | ***% / Mean (±SD)*** | ***% / Mean (±SD)*** |
| Gender (% boys) |  | 1676 | 0 (0%) | 47.4% | 47.7% | 47.3% | 47.2% |
| Age (years) |  | 1676 | 0 (0%) | 7·5 (±2·16) | 7·6 (±2·16) | 7·4 (±2·22) | 7·6 (±2·13) |
| Ethnicity (% Western)^¥^ | | 1016 | 660 (39.4%) | 94.1% | 93.0% | 96.0% | 93.4% |
| SES (%) | *Lowest tertile* | 1673 | 3 (0.2%) | 31.3% | 31.8% | 15.9% | 42.1% |
|  | *Middle tertile* | ·· | | 34.5% | 29.2% | 43.4% | 32.4% |
|  | *Highest tertile* | ·· | | 34.2% | 39.1% | 40.8% | 25.5% |
| BMI z-score |  | 1109 | 567 (33.8%) | 0·135 (±1·02) | 0·051 (±1·01) | 0·092 (±0·95) | 0·232(±1·07) |
| BMI | Total | 1109 | 567 (33.8%) | 17·04 (±2·55) | 16·80 (±2·39) | 16·85 (±2·31) | 17.37 (±2.81) |
|  | Age 5-7 | ·· | | 16.15 (±1.78) | 16·23 (±1·85) | 16·15 (±1·58) | 16·10 (±1·90) |
|  | Age 8-10 | ·· | | 17.41 (±2.67) | 16·84 (±2·32) | 17·02 (±2·26) | 18·08 (±3·00) |
|  | Age 11-13 | ·· | | 18.28 (±3.10) | 18·32 (±3·27) | 18·27 (±3·20) | 18·27 (±2·92) |
| Overweight & obesity ^*^ | Total | 1109 | 567 (33.8%) | 19.9% | 16.5% | 17.9% | 24.1% |
| Overweight ^*^ |  | ·· | | 15.9% | 13.1% | 16.2% | 17.7% |
| Obesity ^*^ |  | ·· | | 4.0% | 3.4% | 1.7% | 6.4% |

**Notes:** BMI = body mass index, HPSF = Healthy Primary School of the Future, PAS = Physical Activity School, SD = standard deviation, SES = socioeconomic status.
^¥^ Information on children’s ethnicity was collected from annual parental questionnaires. Ethnicity, being native background, Western background or a Non-Western background was based on the country of birth of both parents. Ethnicity was subsequently divided into Western (including native background) and non-Western background [15]. ^*^ International Obesity Task Force (IOTF) cut-off values for childhood overweight and obesity.
