# Supplementary material for: Lifetime cost-effectiveness and equity impacts of the Healthy Primary School of the Future initiative
Source: BMC Public Health. 2020 Dec 9;20:1887. doi: 10.1186/s12889-020-09744-9 (PMC7724829; doi:10.1186/s12889-020-09744-9)
Supplement: Supplementary file 3 — Additional file 3. [file 12889_2020_9744_MOESM3_ESM.docx]

**Additional File 3. Details and assumptions pertaining to the childhood and adolescence model**

1. **Assumptions for model input parameters**

**Population.** All 4-12 year-olds in the Netherlands are enrolled at primary schools and exposed to HPSF and PAS. In order to calculate the average QALYs and costs for a school cohort of primary school-aged children (4-12 year olds), the results were aggregated based on the number of Dutch 4-12 year-olds (2019).^1^

**Relative intervention effects.** Information on children’s BMI development was obtained from a previous study.^2, 3^ In the probabilistic analysis, a negative gamma distribution was used to ensure that the relative effects on BMI range from zero to minus infinity (and thus reflect reductions in BMI for HPSF and PAS versus control schools). In a sensitivity analysis this assumption is relaxed by assuming a normal distribution. BMI values were converted into weight categories by using the Dutch reference values for the skewness and variation of the childhood BMI distribution and the age- and sex-specific international cut-off points (IOTF) for childhood overweight and obesity.^4, 5^ **Intervention costs.** The intervention costs were estimated for a future steady state and were informed by expert opinion.^6^ In the calculation of the intervention costs from a societal perspective, the duration of the extended school day was valued as a productivity gain for caregivers as they could spent this time on work. For the cost-effectiveness calculations under the healthcare perspective, this productivity-related cost offset was not included.
 **Health-related quality of life.** HRQOL weights were obtained from Brown et al. (2018), who meta-analysed preference-based utility values per weight category for 5-18 year olds.^7^ It was assumed that the HRQOL effects associated with overweight and obesity were perceived by all 4-20 year-olds and remained constant over time. In a sensitivity analysis, the HRQOL effects were not applied during the primary school period, based on the inconclusive findings on the HRQOL effects for young children.^7^ Analysis of EQ5D-Y data in our study sample (baseline data), however, showed larger differences for children younger than 8 years (scored by parental proxies) as compared to older peers (scores by children themselves).

**Healthcare costs.** Inputs for excess healthcare costs associated with overweight and obesity were obtained from Gortmaker et al. (2015), and combined with the number of healthcare visits and standard cost prices for children in the Netherlands. ^8-10^ We assumed that the relative risks for the impact of overweight and obesity on healthcare costs were are applicable for all 4-20 year-olds and remained constant over time. The healthcare costs were assumed to be fixed, because the data was based on a large study (*Gezondheidsenquete, random sample of the Dutch population, N=9500)* and because variance parameters could not be obtained.

**Productivity costs.** The excess missed school days associated with overweight and obesity were obtained from the meta-analysis by An et al. (2017) and were combined with the number of missed schooldays for children with a healthy weight and the Dutch shadow price for school absenteeism. ^11-13^ It was assumed that the education costs of primary schools in the Netherlands represented (shadow price) the 'soft costs' associated with absenteeism from school. These costs were applied to both the primary and secondary school period.

**Effect maintenance.** In the constant exposure-effect scenario, we used the 2-year observed intervention effects instead of the probability distributions obtained from expert opinion. This was done in order to be able to model the SES-specific impacts for this scenario. The probability distributions defined by experts were also close to the analysed 2-year effects, because experts anticipated that the 2-year intervention effects would (more or less) remain constant over the primary school period. ^3^ For the household multiplier scenario in the primary school period, the 2-year relative effects were first applied, after which the multiplier scenario started (from year 3 onwards).

**Equity impacts.** The HRQOL effects, healthcare and productivity costs were held constant over the different SES groups, based on the assumption that the impacts of overweight and obesity on HRQOL and costs were equal for low, middle, and high SES groups.

**B. Structural model uncertainty**

The analysis adopted a societal perspective. Although intervention costs were assessed from a societal perspective, and both healthcare and productivity costs were included, it may not reflect a *full* societal perspective. Other potential impacts are:

- Wellbeing losses experienced by caregivers due to the forgone caregiver time as result of the extended school day at HPSF.
- Stigmatization/ bullying and wellbeing effects related to overweight and obesity and related to the interventions at HPSF and PAS.
- Wellbeing effects (effects not fully captured with the EQ5D-Y instrument) due to improved lifestyle behaviours.
- The impact of improved lifestyle behaviours on school behaviours and school outcomes: e.g. concentration, cognitive and non-cognitive functioning.

**REFERENCES**

1. Statline. Bevolking; geslacht, leeftijd en burgerlijke staat, 1 januari 2019. <https://opendata.cbs.nl/statline/#/CBS/nl/dataset/7461BEV/table?fromstatweb>. Accessed January 2020.
2. Bartelink N, Van Assema P, Kremers, et al. Can the Healthy Primary School of the Future offer perspective in the on-going obesity epidemic in young children? – a quasi-experimental study. BMJ Open. 2019;9:e030676.
3. Oosterhoff M, Jolani S, De Bruijn-Geraets D, et al. BMI trajectories after primary school-based lifestyle intervention: unravelling an uncertain future. A mixed methods study. Submitted. 2020.
4. Cole TJ, Bellizzi MC, Flegal KM, Dietz WH. Establishing a standard definition for child overweight and obesity worldwide: international survey. BMJ. 2000;320(7244):1240-3.
5. Schonbeck Y, Talma H, van Dommelen P, et al. Increase in prevalence of overweight in Dutch children and adolescents: a comparison of nationwide growth studies in 1980, 1997 and 2009. PloS One. 2011;6(11):e27608.
6. Oosterhoff M, Bosma H, van Schayck OCP, Joore MA. A Cost Analysis of School-Based Lifestyle Interventions. Prev Sci. 2018;19(6):716-27.
7. Brown V, Tan EJ, Hayes AJ, Petrou S, Moodie ML. Utility values for childhood obesity interventions: a systematic review and meta-analysis of the evidence for use in economic evaluation. Obes Rev. 2018;19(7):905-16.
8. Gortmaker SL, Wang YC, Long MW, et al. Three Interventions That Reduce Childhood Obesity Are Projected To Save More Than They Cost To Implement. Health Aff. 2015;34(11):1932-9.
9. Central Bureau for Statistics (CBS). Gezondheid en zorggebruik. n.d. <https://opendata.cbs.nl/statline/#/CBS/nl/dataset/83005ned/table?fromstatweb>. Accessed January 2020.
10. Zorginstituut Nederland. Kostenhandleiding: Methodologie van kostenonderzoek en referentieprijzen voor economische evaluaties in de gezondheidszorg. 2015. <https://www.zorginstituutnederland.nl/binaries/zinl/documenten/publicatie/2016/02/29/richtlijn-voor-het-uitvoeren-van-economische-evaluaties-in-de-gezondheidszorg/Richtlijn+voor+het+uitvoeren+van+economische+evaluaties+in+de+gezondheidszorg+%28verdiepingsmodules%29.pdf>. Accessed January 2020.
11. An R, Yan H, Shi X, Yang Y. Childhood obesity and school absenteeism: a systematic review and meta-analysis. Obes Rev. 2017;18(12):1412-24.
12. Drost RMWA, Paulus A, Ruwaard D, Evers S. Handleiding Intersectorale Kosten en Baten van (Preventieve) Interventies. Maastricht University, Faculty of Health, Medicine and Life Sciences CAPHRI, School for Public Health and Primary Care Care, Department of Health Services; 2014.
13. Willeboordse M, Jansen MW, van den Heijkant SN, et al. The Healthy Primary School of the Future: study protocol of a quasi-experimental study. BMC Public Health. 2016;16(1):639.
