# Supplementary material for: Lifetime cost-effectiveness and equity impacts of the Healthy Primary School of the Future initiative
Source: BMC Public Health. 2020 Dec 9;20:1887. doi: 10.1186/s12889-020-09744-9 (PMC7724829; doi:10.1186/s12889-020-09744-9)
Supplement: Supplementary file 4 — Additional file 4. [file 12889_2020_9744_MOESM4_ESM.docx]

**Additional File 4. Details and assumptions pertaining to the adulthood model**

1. **Assumptions for model input parameters**

**Chronic disease model.** The RIVM Chronic Disease Model (CDM), a probabilistic health economic model with the Markov property, estimates the prevalence, incidence, and mortality of major chronic diseases based on changes in risk factors. ^1^ Risk factors for which the impact on chronic diseases can be modelled are, for example, smoking, body mass index, physical activity, and fruit and vegetable intake. In the CDM, body weight is defined by three classes: normal weight, overweight (BMI 25-30 kg/m2), and obesity (BMI ≥30 kg/m2).^2^

**Chronic diseases.** Obesity-related diseases in the CDM include myocardial infarction (AMI), angina pectoris, chronic heart failure, stroke, renal, colorectal, breast, prostate, and endometrium cancer, diabetes mellitus, hip and knee arthritis, and low back pain. The CDM also describes the independent effect of diseases on other diseases (i.e. persons with diabetes have a higher risk of myocardial infarction compared to persons without diabetes, independently from overweight). ^1^ In addition, changes in non-overweight related diseases may occur during life years gained. This may be caused by interventions that result in an increase of life years, which are not likely to be lived in full health (i.e. incidence of lung cancer during added life years in case of obesity prevention). Indirect related diseases are chronic obstructive pulmonary disease (COPD), lung, stomach, esophagus, larynx, bladder, pancreas, and oral cavity cancer. ^3^

**Utilities.** The health state utilities in the CDM were obtained from the Dutch Burden of Disease Study containing utilities for several chronic diseases. ^4^ The health state utilities for overweight and obesity are not included. Therefore, gains in quality of life result from decreases in the prevalence of chronic diseases, and not from weight loss only. Proportional utility weights for QALYs were used in the CDM, which implies that the disability of comorbidity increases with the number of diseases present, however this effect is less than additive. ^3^

**Healthcare costs.** The model used data of the Dutch Cost of Illness Study for the Dutch healthcare costs.^5^ Cost data were indexed to the Dutch 2018 price level using consumer price indexes.

**Discounting.** Because obesity prevention was applied in the primary school period, we further discounted the healthcare costs and effects over 12 years, spanning the time from childhood (mean 8 years of age) into young adulthood (20 years of age).

**Productivity costs.** Productivity losses in adulthood were incorporated through the relation between weight category up to 67 years of age and the number of annual sick leave days from work as reported by Lehnert et al. (2014). ^6, 7^

**B. Structural model uncertainty**

The analysis adopted a societal perspective. Although both healthcare and productivity costs were included, it may not reflect a full societal perspective. Other potential impacts are:

- Stigmatization and wellbeing effects related to overweight and obesity.

- Wellbeing effects (effects not fully captured with the EQ5D-Y instrument) due to improved lifestyle behaviours.

- The impact of improved lifestyle behaviours on school outcomes and productivity in later life.

**REFERENCES**

1. Hoogenveen RT, van Baal PH, Boshuizen HC. Chronic disease projections in heterogeneous ageing populations: approximating multi-state models of joint distributions by modelling marginal distributions. Math Med Biol. 2010;27(1):1-19.
2. Schonbeck Y, Talma H, van Dommelen P, et al. Increase in prevalence of overweight in Dutch children and adolescents: a comparison of nationwide growth studies in 1980, 1997 and 2009. PloS One. 2011;6(11):e27608.
3. van Baal PHM, Hoogenveen RT, de Wit GA, Boshuizen HC. Estimating health-adjusted life expectancy conditional on risk factors: results for smoking and obesity. Popul Health Metr. 2006;4(1):4-14.
4. Melse JM, Essink-Bot ML, Kramers PG, Hoeymans N. A national burden of disease calculation: Dutch disability-adjusted life-years. Dutch Burden of Disease Group. Am J Public Health. 2000;90(8):1241-7.
5. Slobbe L, Smit J, Groen J, Poos M, Kommer G. Kosten van Ziekten in Nederland 2007.
6. Lehnert T, Stuhldreher N, Streltchenia P, Riedel-Heller S, Koenig H-H. Sick leave days and costs associated with overweight and obesity in Germany. J Occup Eviron Med. 2014;56(1):20-7.
7. CBS Statline. Arbeidsdeelname. 2017. <https://opendata.cbs.nl/statline/#/CBS/nl/dataset/71738NED/table?fromstatweb>]. Accessed January 2020.
