# Supplementary material for: Lifetime cost-effectiveness and equity impacts of the Healthy Primary School of the Future initiative
Source: BMC Public Health. 2020 Dec 9;20:1887. doi: 10.1186/s12889-020-09744-9 (PMC7724829; doi:10.1186/s12889-020-09744-9)
Supplement: Supplementary file 5 — Additional file 5. [file 12889_2020_9744_MOESM5_ESM.docx]

**Additional File 5. Sensitivity analyses**

**Univariate sensitivity analysis**

- No overall effects were found in the analysis of baseline HRQOL data from the quasi-experimental study (EuroQol-5D Youth instrument). Similarly, Brown et al. (2018) report that the evidence on the impact of overweight and obesity on HRQOL in young children is scarce and inconclusive. ^1^
- Ratio of healthcare costs for overweight and obesity vs. normal weight based on the quasi-experimental study. For obese children who incurred any healthcare costs, we found a rate ratio of 1.87 for general practitioner related healthcare consumption and a rate ratio of 1.59 for specialist visits as compared to normal weight children.
- Ratio of annual school absenteeism days for overweight and obesity vs. normal weight based on the quasi-experimental study. We found a ratio 1.21 for overweight children and a ratio of 1.59 for obese children as compared to children with a normal weight.
- HRQOL determined by weight status instead of by chronic disease in adulthood. The HRQOL benefits (for intervention versus control) are accrued later in time when they are exclusively linked to the occurrence of chronic disease as when they are related to weight status directly. For this, we used a disutility of 0.017 per unit increase in BMI and assumed and an average 5-unit BMI difference between the weight classes (normal weight, overweight and obesity). ^2^
- Time horizon till age 70 years.
- Short-term cost of HPSF and PAS (at year 1 and year 2) instead of the estimated long-run costs only. ^3^
- Discount rates of 3% for both costs and effects.

**Probabilistic sensitivity analyses**

Childhood and adolescence model

Probabilistic analyses were run for every age-cohort. It was assumed that the number of simulations per age cohort and the limited number of probabilistic input parameters mitigated the potential influence of the differences in random draws on cost-effectiveness results.

Adulthood model

A probabilistic sensitivity analyses was conducted to account for the uncertainty in input parameters. The intervention effect, prevalence rates of weight class in adulthood, and the disease prevalence and incidence were included as probabilistic parameters. The uncertainty of the intervention effect was incorporated by including the overweight and obesity prevalence rates at young adulthood as probabilistic parameters. This uncertainty parameter reflected the boundaries of the 95% confidence interval of the intervention effect on body mass index. The overweight and obesity prevalence rates at 20 years of age were included as multivariate normal distributions with a perfect correlation. Results were analysed as the mean of 100 replications based on random draws of the probabilistic parameters. We would have preferred to use an increased number of random draws in the adult model, but that would have increased computation time tenfold for 1000 instead of 100 draws, which would have become unworkable. The uncertainty intervals for both costs and effects did overlap when running another 100 PSA draws in the adulthood model, which suggests that the PSA results were stable.

Results were summed up, assuming independence between the probabilistic results of the childhood and adulthood model.

**REFERENCES**

1. Brown V, Tan EJ, Hayes AJ, Petrou S, Moodie ML. Utility values for childhood obesity interventions: a systematic review and meta-analysis of the evidence for use in economic evaluation. Obes Rev. 2018;19(7):905-16.
2. Hakim Z, Wolf A, Garrison LP. Estimating the effect of changes in body mass index on health state preferences. PharmacoEconomics. 2002;20(6):393-404.
3. Oosterhoff M, Bosma H, van Schayck OCP, Joore MA. A Cost Analysis of School-Based Lifestyle Interventions. Prev Sci. 2018;19(6):716-27.
